# Antimalarial, Antioxidant Activities and Chemoprofile of *Sansevieria liberica* Gerome and Labroy (Agavaceae) Leaf Extract

**DOI:** 10.1155/2021/9053262

**Published:** 2021-12-06

**Authors:** Ifeoma Celestina Orabueze, Sarah Chiamaka Uzor, Bou Ndiaye, Desire Uba, Duncan Azubuike. Ota, Joseph Agbedahusi

**Affiliations:** ^1^Departments of Pharmacognosy, Faculty of Pharmacy, College of Medicine, University of Lagos, Surulere, Lagos, Nigeria; ^2^Laboratoire Eau Energie Environnement et Procédés Industriels (LE3PI) Ecole Supérieure Polytechnique de Dakar (ESP), Université Cheikh Anta Diop de Dakar, Dakar, Senegal; ^3^Center for Studies on Food Safety and Functional Molecules (CESAM-RESCIF) ESP-UCAD, Dakar, Senegal; ^4^Department of Physiology, College of Medicine, University of Lagos, Surulere, Lagos, Nigeria; ^5^Drug Research and Production Unit, Faculty of Pharmacy, Obafemi Awolowo University, Ife, Nigeria

## Abstract

**Background:**

*Sansevieria liberica* Gerome and Labroy (Agavaceae) is a religious and ornamental perennial plant with highly valued medicinal usage in Nigeria. Sansevieria liberica is used in the management of malarial fever. The ease of development of resistance to available antimalarial drugs has resulted in increased clinical failure and mortality. The study investigated the antimalarial effects of *Sansevieria liberica* (SL) leaf extract in mice infected with *Plasmodium berghei*.

**Materials and Methods:**

The ability of SL leaf extract to suppress the growth of malaria parasites in early (suppressive) and established (curative) infections was established using animal models. The mean survival time (MST) was determined. The antioxidant potential was established using two standard *in vitro* models. High-performance liquid chromatography (HPLC) and phytochemical analysis methods were used to create a chemoprofile fingerprint of SL hydroethanolic leaf extract.

**Results:**

At 200, 400, and 400·mg kg^−1^, SL produced 68.71, 70.74, and 75.09% parasite suppression in the suppressive model while the curative model gave a percentage of cure of 71.09, 72.60, and 62.09, respectively. The animals lived longer compared to both negative and positive controls but were not fully protected. The IC_50_ values of SL and vitamin C were calculated to be 3.599 *µ*g mL^−1^ and 3.08 *µ*g mL^−1^, respectively. The reducing power of vitamin C was significantly (*P* < 0.05) higher than that of SL extract. Some flavonoids were established as possible marker compounds for SL leaf extract. *Discussion and Conclusions*. The antimalarial assay results demonstrated that the use of SL in folk medicine may have scientific support.

## 1. Introduction

*Sansevieria liberica* Gerome and Labroy (Agavaceae) is a religious and ornamental perennial plant with dark green, usually paler indistinct, transverse bands arising from the rhizome. It is widely distributed in West Africa. It is commonly seen along the waterway and thick forest. In the Southeast of Nigeria, it has a religious orientation and is believed to be a plant of the gods [1]. It is normally seen in worship places. A lot of myths are attached to the plant, and collection of the plant was restricted in the past due to religious beliefs. It is also grown for its fibers. It is called “ebube agu” in the Southeast of Nigeria, which literally can be interpreted as the “aura of a lion” or the “aura of a thick forest.” The implication of this name means that it is a plant with “fear and respect” attached to it. *Sansevieria liberica* (SL) is a medicinal plant that is seen in almost every private medicinal garden of the traditional healers due to its acclaimed efficacy in the management of many common and uncommon illnesses. Other local names for SL in Nigeria include Ola-koriko (Yoruba), Mooda (Hausa), African bowstring hemp, Leopard lily, mother-in-law's tongue, and devil's tongue [2].

Different morphological parts of SL are used in the traditional healing system in Nigeria but the leaves and roots are of greater ethnomedicinal importance. Some of these uses include management of malarial symptoms (fever, headache, and cold, as well as an analgesic), anti-infective, sedative, anticonvulsant, anti-inflammatory agent, abortifacient, and being administered during labor [1]. Other reported uses of the leaves and roots include treatment of asthma, abdominal pains, colic, diarrhea, eczema, gonorrhea, hemorrhoids, hypertension, menorrhagia, piles, sexual weakness snake bites, and wounds of the foot [3, 4]. Herbal preparations made from the leaves of SL are aged recipes in the traditional healing of malaria fever, convulsion, and pain [3]. Some of the ethnomedicinal claims of efficacy of SL have been evaluated scientifically, and the findings were published in various journals. Reference [2] reported that the aqueous root extract of SL is an effective antidiarrhoeal agent. The antisnake venom, sedative, asthma, diabetes, abdominal pains, anticancer, hepatoprotective, CNS depressant, anticonvulsant, analgesic, anti-inflammatory and anticonvulsant, antidiabetic, antileishmanial, and antiplasmodial activities of SL have been evaluated and reported by various research teams [5–11].

Malaria is caused by *Plasmodium* spp. and transmitted by the vector *Anopheles* mosquito during bite while seeking a blood meal. The Plasmodium while in the body causes lots of changes to the wellbeing of the individual. It breaks down the red blood cells causing malaria-oriented anemia and releases toxins into the blood which causes fever. Other symptoms of malaria include vomiting, headache, and chills. Malaria is a debilitating disease with a high mortality and morbidity rate [12]. It has a huge negative effect on health and socioeconomic impact [13]. The incidence of malaria is more pronounced in poor countries within the sub-Sahara. The most affected populations are the pregnant women due to their compromised health status and the children under the ages of 5, who are yet to develop immunity against the *Plasmodium*.

Global and local efforts have been made to control and/or eradicate the infection without much success. The failure has been attributed to the multidrug-resistant effect of the *Plasmodium* to available drugs, ease of development of resistance to insecticides by the vector, and challenges in the implementation of national malaria control policies. Early diagnosis and initiation of efficacious antimalarial drugs are crucial in the management of the infection. Proper clinical treatment of malaria infection involves total clearance of the parasite from the blood and liver system to prevent recrudescence, preventing the reintroduction of malaria, driving down transmission that could be caused by reservoir patients (asymptomatic persons, having the parasite but not showing any symptoms or signs of the infection [14]. The nonavailability of efficacious antimalarial drugs in an endemic area may prove to be a crucial health challenge considering the high rate of death and infection as annually announced by the World Health Organization (WHO). The global death due to malaria for 2019 was estimated to be about 409,000 by WHO. Africa region was responsible for about 94% of this malaria burden figure. World Health Organization [15] reported that Nigeria accounted for 27% of the global malaria incidence and the highest number of resulting death due to malaria (23%) for the year 2019 [16].

Medicinal plants consist of many bioactive components which have always been found useful in the management of health issues. The medicinal plants are either used as whole extracts or semipurified fractions or individual compounds or modified isolated compounds. Before initiation of conventional drugs, treatment of malaria fever was dependent heavily on herbal or traditional herbal preparations and home remedy outcomes. An increase in oxidative stress has been reported to negatively affect cellular integrity and could lead to malaria complications [17].

Malaria has been long treated with drugs of plant origin and their derivatives such as quinine, chloroquine (an analog of quinine), and currently artemisinin. The ease of emergence of resistance to available antimalarial drugs necessitates the urgent need and continuous search for new antimalarial drugs. There have been reports of resistance with the clinically current and WHO-approved artemisinin combination therapy (ACT) indicating that the level of malaria infection threat will be on the rise if nothing is done urgently.

The current study aimed to evaluate the antimalarial effect of SL and to confirm the viability of its use in the treatment of malaria in folk medicine. The study also aimed at the discovery and development of drug candidates from SL leaf extract with desirable characteristics such as good efficacy, safe, possibly new mechanism of activity, and good stability properties when used in a formulation. The antioxidant value and chemical fingerprint of the plant were also studied.

## 2. Materials and Methods

### 2.1. Plant Material Collection

The fresh leaves of *Sansevieria liberica* (SL) were collected from Nsukka, a town in the Southeast part of Nigeria in March 2016. The leaves were identified by Mr. Ozioko, Fred. He is a taxonomist at the International Centre for Ethnomedicine and Drug Development (InterCEDD) where a herbarium specimen was deposited. The voucher number of InterCEDD/1605 was assigned to *Sansevieria liberica.*

The leaves were washed, sliced to smaller sizes, and air-dried for three weeks at room temperature.

### 2.2. Extraction

The dried pieces of SL were powdered and weighed (318 g). The pulverized plant material was extracted employing the cold maceration technique. It was soaked in 3 L of 90% methanol. Maceration was done for 72 h, and manual stirring was done twice daily. Filtration was done using a doubled muslin cloth. The resultant residue was remacerated for another 72 h and filtered. The obtained filtrates of the hydroalcoholic extract were concentrated using Buchi Rotavapor at a preset temperature of 40°C. Drying was completed between a regulated water bath at 40°C and a desiccator. The dried extract obtained was weighed, transferred into McCartney bottles, labeled appropriately, and stored in a deep freezer. The yield was 62 g (19.5%).

### 2.3. Phytochemical Test

The crude leaf extract of SL was screened for the presence of secondary metabolites using standard procedures [18, 19].

### 2.4. Animals

A total of fifty albino mice with a weight range of 18–22 g were used for the *in vivo* assays for antimalarial activities of SL. They were maintained in the animal house of the University of Lagos, College of Medicine, Idi-Araba, where they were kept in separate plastic cages with a metal cover for the free passage of air. The animals were acclimatized for 7 days before the commencement of the experiments. Dark and light cycles were maintained at 12 h each.

### 2.5. *Plasmodium berghei*

A mouse infected with chloroquine-sensitive NK-65 strain *Plasmodium berghei* was obtained from the National Institute of Medical Research (NIMR) Yaba Lagos Nigeria. The rodent parasite was maintained by subpassage in an uninfected mouse at 7-day intervals.

### 2.6. Acute Toxicity Test

Acute *in vivo* toxicity was performed using the method of Lorke's [20], and the median lethal dose (LD_50_) was determined. The animals were divided into dosing groups of 6 mice per group. Four different dose levels of SL leaf extract were administered orally to the animals. Each group received a particular dose level. The 4 different treatment doses of SL extract were 500, 1000, 2000, and 4000 mg·kg^−1^. The untreated group (negative control) was given the vehicle, 5% DMSO. The animals were kept under watch for any sign of distress or death.

### 2.7. Suppressive Antimalarial Bioassay of SL Extract against *P. berghei*

The suppressive effect of SL leaf extract was carried out using the 4-day suppressive method as described by Orabueze et al. [21] and Peters et al. [22]. All the experimental animals were injected with 1 × 10^7^*P. berghei* parasitized erythrocytes on the first day (*D*_0_). They were randomly grouped into 3 different treatment groups and 2 control groups. Each group consists of 5 animals. Oral administration of the crude extract was initiated 2 h after parasite inoculation. Groups 1, 2, and 3 received 100, 200, and 400 mg·kg^−1^ of SL leaf crude extract, respectively, in 0.2 mL volume. Control groups 4 and 5 received 10 mg·kg^−1^ of chloroquine and 5% DMSO, respectively, in 0.2 mL volume. The drugs were administered orally, as single daily doses for four consecutive days (*D*_0_—*D*_3_). And on the fifth day (*D*_4_), blood was taken from the tail of each mouse and used in preparing a thin blood smear. These were fixed with ethanol and stained with Giemsa stain. The antimalarial activity or level of parasitemia was determined by examining the thin stained blood smear under the microscope.

The average percentage parasitemia for each smear was determined.

(1) $$\begin{array}{c} \frac{{}_{p}R\text{.}B\text{.}C}{{}_{T}R\text{.}B\text{.}C}\times 100, \end{array}$$

where _*p*_RBC is the number of parasitized red blood cells and _*T*_RBC is the total number of red blood cells in view, and the percentage parasitemia chemosuppression (suppression) for each dose was calculated as

(2) $$\begin{array}{c} \frac{\left(N-T\right)}{N}\times 100, \end{array}$$

where *N* is the average percentage of parasitemia in the negative control group and *T* is the average percentage of parasitemia in the test groups.

### 2.8. Curative Antimalarial Bioassay of SL Extract against *P. berghei*

The curative antimalarial activity (also known as Rane's test) of hydroethanolic leaf crude extract of SL was carried out according to the method of Ryley and Peters [23] with slight modification. The experimental mice were injected i.p with chloroquine-sensitive NK- 65 strain of *P. berghei* on the first day (*D*_0_). The mice were left untreated for 72 h for the infection to be established. The presence of infection and level of parasitemia was confirmed by a thin blood smear prepared from the tail blood of each animal. The volume of parasitemia after 72 h (*D*_3_) served as the basal parasitemia load. The mice were randomly divided into 3 treatment groups and 2 control groups of five mice each. The treatment groups 1, 2, and 3 were, respectively, administered orally with graded doses (100, 200, and 400 mg·kg^−1^) of the SL leaf extract test drug. The control groups 4 and 5 received chloroquine 10 mg·kg^−1^ and 5% DMSO in 0.2 mL volume, respectively. The drug was administered daily for 5 days, from *D*_3_ to *D*_7_. A thin blood smear was prepared from each of the animals for each treatment day to monitor the rate of parasite reduction or clearance parasite from the blood system of the mice. A final thin blood smear was prepared on *D*_8_, that is, 24 h after the last dose to determine the parasitemia reduction contributory effect of the dose received on *D*_7_. The percentage of cure or curative effect or percentage of inhibition was calculated.

The animals were allowed to live, and any death from any group was recorded and used to calculate the mean survival time (MST) for each group. This was done by finding the average survival time (days) of the mice (after inoculation) in each group over a period of 22 days (*D*_0_—*D*_21_).

(3) $$\begin{array}{c} \frac{\text{MST}=\text{sum of survival time of all mice in a group daily}}{\text{total number of mice in the group}}. \end{array}$$

### 2.9. Antioxidant Assays

#### 2.9.1. DPPH Scavenging Activity

The free radical scavenging potential of SL leaf extract was evaluated using the stable 1, 1-diphenyl-2-picryl-hydrazyl (DPPH) radical scavenging assay as described by Mensor et al. [24] with some modifications. Various dilute serial concentrations of SL hydroethanolic leaf extract were prepared with methanol. To 1 mL of each prepared dilute concentration of SL extract, 4 mL of methanol and 1 mL of DPPH (100 *µ*M methanolic solution) were added, and the solution was left in a dark place at room temperature to incubate for 30 min. And absorbance was recorded at 517 nm. Vitamin C was used as a standard, and its DPPH radical scavenging assay was also done using serially diluted concentrations. Measurements were done in triplicate.

### 2.10. Reducing Power Assay

The reducing power of hydromethanolic (90%) leaf extract of SL was evaluated using the method described by Yen and Chen [25]. The reference drug used for the study was vitamin C (ascorbic acid) while the negative control was a blank (solvent used for the constitution but contained no drug). Serial dilutions of both the test SL extract and the reference were made with methanol. From each of the different diluted concentrations of the SL, 1 mL was measured and mixed with 2.5 mL of 0.2 M sodium phosphate buffer (pH 6.6) and 2.5 mL of 1% potassium ferricyanide. The resultant mixtures were incubated for 20 min at 50°C, and 2.5 ml of 10% trichloroacetic acid was added to each of them and centrifuged for 10 min. A volume of 2 mL of the resulting supernatants was mixed with 0.4 mL of freshly prepared ferric chloride (FeCl_3_ 0.1%, w/v) and 2 mL of deionized water. Absorbance was measured at 700 nm after 10 min of mixing and incubation. The above method was repeated using the reference drug, ascorbic acid. Each determination was done in triplicate.

An increase in absorbance power is an indication of an increase in reducing power.

### 2.11. High-Performance Liquid Chromatography (HPLC) Analysis

HPLC analysis was performed using Agilent Technologies HPLC 1200 series, equipped with RP-18 column (150 mm × 4.6 mm, 5 *µ*m particle size, Merck) and Agilent UV detector. Acetonitrile HPLC grade and purified water containing (0.1% formic acid) were used as the mobile phase at the ratio (50 : 50) v/v. An isocratic system of elution was employed and a run time of 45 min. The SL crude extract and the reference standards were prepared in methanol, at a concentration of 10 mg/5 mL and 200 *µ*g/mL, respectively. Analysis was performed using a flow rate of 1.0 mL/min and monitored at 280 nm wavelength. The temperature was maintained at 20–25°C for all chromatographic operations. The identification of the peaks was done by comparing their retention time with that of the standards and UV absorption spectrum of authentic reference standards. The reference samples used were gallic acid, catechin, rutin, quercetin, naringenin, and kaempferol.

### 2.12. Statistical Analysis

Data obtained from the study are presented as mean ± SEM. Graphpad version 5.00 was used to analyze the data. Statistical significance was determined by one-way ANOVA followed by Tukey's technique to compare results between treatment and control groups. For all the data obtained, the result was considered significant at *P* value <0.05.

## 3. Results

### 3.1. Acute Toxicity Test

No death or observable physical distress was noticed at the highest dose of 4000 mg·kg^−1^ used for the toxicity testing. It was not possible to calculate the LD_50_ since no death occurred at this level of dosing. The various dose levels of SL hydromethanolic leaf crude extract produced no immediate or delayed distress on the animals or any visible behavioral changes, thus, suggesting that the experimental doses used for the study can be considered to be safe.

### 3.2. Suppressive Activity of SL Extract against *P. berghei*

**Figure 1:**
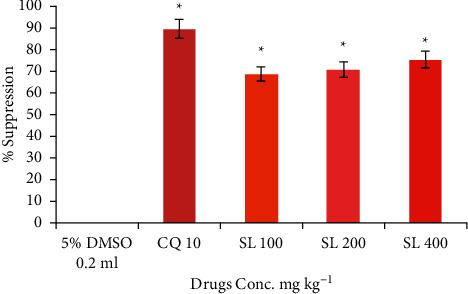
Suppressive effect of 90% methanolic leaf extract of SL in early infection of *Plasmodium berghei* Values for mean % parasitemia are expressed as mean ± standard error of the mean (±SEM) *n* = 5, significant at ^*∗*^*P* < 0.05 when compared with the control. SL: *Sansevieria liberica* extract CQ: chloroquine

The results of early infection of the leaf extract are shown in Figure 1 (Suppl. 1). The *P. berghei* based screening showed a significant dose-dependent chemotherapeutic effect compared to the negative control (*P* < 0.05). Chemosuppression effects of the various doses, 100, 200, and 400 mg·kg^−1^ of the extract were 68.71, 70.74, and 75.09%, respectively.

The standard drug chloroquine gave a chemosuppression of 89.36%, which is significantly higher than the effect of the treatment's highest dose of 400 mg·kg^−1^. The reductions in parasitemia levels of the treated groups were statistically significantly low (*P* < 0.05) compared to the negative control group that received only the vehicle (5% DMSO).

### 3.3. Curative Activity of SL Extract against *P. berghei*

The SL leaf extract showed schizonticidal potential across the three treatment groups (100, 200, and 400 mg·Kg^−1^). However, the effect of the parasite clearance is not dose-dependent (Table 1). The decrease in the percentage parasitemia exhibited at 400 mg·kg^−1^ of the extract is low compared to the percentage reduction obtained at 100 and 200 mg·kg^−1^. The percentage reduction (cure) obtained for the study was 71.09, 72.60, and 62.09% for 100, 200, and 400 mg·kg^−1^ respectively on *D*_8_.

These were significantly low compared to the positive control (CQ) but significantly higher compared to the negative control (*P* < 0.05). All the treatment groups and the CQ group exhibited an early onset of activity, with the CQ group experiencing higher parasite reduction in the blood system. The daily increase in parasite clearance was sustained across the groups that received the extract at various doses and the CQ treatment group. However, a drop was observed between *D*_7_ and *D*_8_ of the group that received 400 mg·kg^−1^ of SL extract.

## 4. Antioxidant

**Figure 2:**
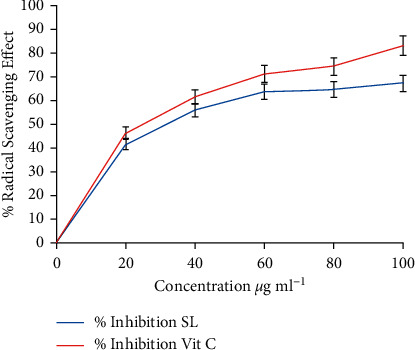
DPPH radical scavenging activities of SL and Vit. C SL: *x* = 3.599; *R*^2^ = 0.7396. Vit. C *x* = 3.082; *R*^2^ = 0.8131.

**Figure 3:**
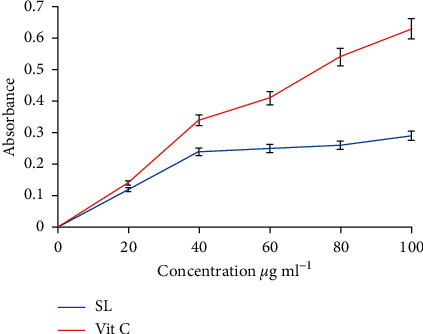
Reducing power of SL and Vit C Vit. C *x* = 397.608; *R*^2^ = 0.9809. SL: *x* = 931.197; *R*^2^ = 0.8153.

The antioxidant capability of the extract of SL was examined by employing the DPPH radical scavenging and ferric reducing assays. The results are shown in Figures 2 and 3. The ability of SL extract to scavenge free radicals was concentration-dependent. The 50% or half-maximal inhibitory concentration (IC_50_) values of hydroethanolic leaf extract of SL and vit. C were calculated to be 3.599 *µ*g mL^−1^ and 3.082 *µ*g mL^−1^, respectively (Figure 2). The extract of SL thus exhibited radical scavenging and antioxidant effects [26].

The reducing powers of both extract and vit. C on Fe^3+^ were concentration-dependent. The reducing power effect of SL extract and reference drug were increasing with a concentration in a strongly linear manner (*R*^2^ = 0.8153 and 0.9809 respectively). Increasing absorbance is an indication of increasing Fe (III) reduction effect of the test drug. The reducing power of vit. C was significantly (*P* < 0.05) higher than that of SL extract (IC_50_ of 931.197 and 397.61 *µ*g·mL^−1^ respectively).

## 5. HPLC Profiling

**Figure 4:**
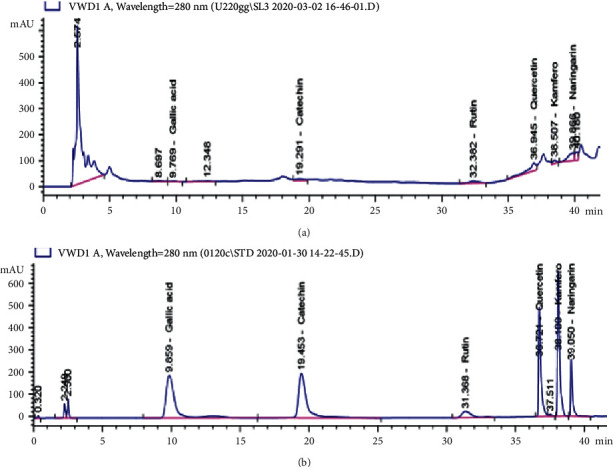
HPLC chromatograms of identified peaks of (a) hydromethanolic leaf extract of SL at 280 nm and (b) a mixture of standard flavonoids at 280 nm.

Hydromethanolic leaf extract of *Sansevieria liberica* showed the presence of six flavonoids, namely, gallic acid, catechin, rutin, quercetin, kaempferol, and naringenin (Figure 4; Table 2). The identification of these compounds was done by comparing their peak information with that of commercial reference standards (Figure 4; Table 3). The highest content of the marker flavonoids was found to be in the order naringenin (18.846 ppm) > rutin (12.777 ppm) > quercetin (9.829 ppm) (Table 2).

## 6. Discussion

The importance of herbal preparations in the management of common health issues in the developing world cannot be overemphasized. Nigeria is a developing country with good biodiversity and over the ages has documented some available plants and their health-food oriented usage. Until recently, *S. liberica* was believed to be a proud and a plant for the gods and wards off evils; thus, its power of healing was assumed to be religious. This traditional belief may be due to its wide range of medicinal uses and healing power and may also be an indirect method of protecting the plant from being overharvested. The medicinal importance of SL in folk medicine has led to its being evaluated and the resultant information documented. The hepatotoxicity profile and safety profile of repeated exposure to aqueous extract of *Sansevieria liberica* were reported by Achi and Ohaeri [3]. Their record showed no evidence of hepatotoxicity or detrimental effect to any of the observed or measured safety parameters. Likewise, mice that received various doses of 90% methanol SL leaf extract in this study showed no observable, physical, or clinical distress. Thus, this result corroborates previous findings on its safety.

The use of herbal preparations for the management of malaria and other diseases has generated a lot of interest especially with the emergence of multidrug-resistant strains of malaria-causing agents. Earlier research within the plant kingdom led to the discovery of the antimalarial properties of *Cinchona officinalis* and *Artemisia annua* [27]. The *in vivo* antimalarial study of the leaf extract of SL was carried out against chloroquine-sensitive *Plasmodium berghei* to understudy its antimalarial potential and a step forward toward sourcing antimalarial chemotherapeutic agents from the plant kingdom. The antioxidant evaluation was included in the study due to the interrelationship between oxidative stress and malaria. In addition, chemoprofiling of the study plant serves as identification and preliminary standardization of the plant and aid during the preformulation study.

The current study was carried out using animal models (*in vivo*) in order to have a system that takes into consideration the prodrug and immune system effects in the course of drug metabolism in a living biological system [28]. The *P. berghei* used in the study is a rodent *Plasmodium* and can produce malaria symptoms similar to human malaria infection caused by *P. falciparum*. The 4-day suppressive and curative (Rane) tests were employed for the antimalarial assays. Each of the antimalarial models has a phase in the malaria fever cycle that it represents. The early-stage malaria was evaluated by the 4-day suppressive test while the established malarial syndrome was evaluated by Rane's curative analysis [29]. The parameters used in assessing the efficacy of the crude drug were the percentage of chemosuppression and inhibition for 4-day suppressive and curative activities, respectively [22, 23]. These methods have been employed in the evaluation of many potential antimalarial agents in several documented studies [30–32].

The results obtained from the antimalarial studies showed that the hydromethanolic leaf extract of SL produced a significant suppressive effect against early *Plasmodium* infection and curative effect against established infection in *P. berghei* infected mice.

The extract produced a significant (*P* < 0.05) dose-dependent chemosuppression in all the treated groups with the highest chemosuppression (75.09%) observed in the group treated with 400 mg·kg^−1^. The dose-dependent pattern of the response may suggest that at an increased dose the suppressive effect of the extract may be higher. Also, there is the possibility of the hydromethanolic leaf extract exhibiting greater activity on purification. The bioactivity of any crude extract that improves with an increase in purification status is an indication of a possible increase in bioactivity on the isolation of the active compounds. Compared with the standard drug, chloroquine, the extract activity of the SL extract is significantly lower. Further analysis of the result showed that there was no significant (*P* > 0.05) difference in the activity between all three doses of *S. liberica.*

The hydromethanolic leaf extract also demonstrated significant daily progressive reduction or decrease of parasitemia across all the groups that received drug treatment (the CQ and extract groups) for the curative test. The CQ group on *D*_7_ showed a total clearance or nondetectable level of the parasite in the blood. Likewise, on *D*_8_, the nondetectable parasite status was maintained indicating that there was an actual total clearance of the parasite. No reservoir was retained. The extract treated groups all showed early onset of activity and a gradual and daily reduction in the volume of the *Plasmodium*; however, none had a total clearance (or 100% cure) as observed with the CQ group. This showed that though the leaf extract of SL can be said to be a promising antimalarial agent, its potency needs to be improved. Traditionally, this may explain the common practice of polyherbal preparations and the long duration of treatment regimes. In polyherbal preparations or practice, the different plant constituents of the product may have synergistic effects or serve different purposes toward increasing the overall efficacy of the drug product and thus facilitate healing. The polyherbal practice may also reduce the use of a large volume of the drug, thus lowering the possibility of toxicity due to large doses. Some of the components that make up polyherbal drugs may act as an analgesic, appetite inducer, blood builder, antipyretic, etc. [21].

The survival time and the percentage inhibition (% cure) at 200 mg·kg^−1^ (72.60%) are higher than those of the treatment group that received 400 mg·kg^−1^ (62.09%). The survival time obtained for 200 mg·kg^−1^also suggested the efficacy of this lower dose compared to 400 mg·kg^−1^. This may be suggesting that the optimal therapeutic dose in mice is within the dose range of 200 mg·kg^−1^. And higher doses of the extract may not possess significantly more beneficial antimalarial effects.

There was an increase in parasitemia between *D*_7_ and *D*_8_ of the group that was treated with 400 mg·kg^−1^ of the extract. This could be an indication that the parasite started regrowing on the immediate withdrawal of the drug (recrudescence). The immediate regrowth of the parasite may be an indication that the extract is fast being metabolized out of the system. The fast recrudescence may also have been triggered by a lowered immunological effect caused by the high dose of the drug. Though the drug may have given a nontoxicity effect in acute toxicity evaluation in the reported case by [3], there may be a need to study long-term and repeated dose toxicity-undetected side effects.

Across the different treatment groups, the animals were protected for 22 days (*D*_21_) in the curative model except for the group that received 400 mg·kg^−1^. This supports the recrudescence noticed on *D*_8_ of the 400 mg·kg^−1^ treatment group, the parasite remultiplying on withdrawal of the drug. The animals that received CQ and the two lower crude test drug dose levels of *S.liberica* extract were alive and active till *D*_21_. There was no significant (*P* > 0.05) difference between 100 and 200 mg·kg^−1^ doses for the curative test. Therefore, 100 mg·kg^−1^ being a lower dose compared to 200 mg·kg^−1^ may be the preferred therapeutic dose because of reduced predispose to side effects. The observed early recrudescence may also be suggesting the need for the addition of another herbal drug (polyherbal preparation) to enhance efficacy and achieve the desired total clearance of the parasite from the body system.

In both models, SL seems to possess the same level of efficacy as a curative and suppressive agent. The research team of [33] has reported the antimalarial (*in vivo*) and antiplasmodial (*in vitro*) effects of methanol extract and dichloromethane fraction of *Sansevieria guineensis* (L.) Willd. collected from Guatemala. The *in vitro* antiplasmodial effect of SL investigated by [6] showed moderate activity due to dichloromethane extract compared to the menthol and aqueous extracts that showed little or no activity. The difference in activity between the study [7] led by a research team and the current study may be due to different modes of extraction and solvents used and assay methods used.

Antioxidant assay protocols used for the study were DPPH and ferric reducing antioxidant power. DPPH (1, 1-diphenyl-2-picrylhydrazyl) radical scavenging assay has been reported to be an effective evaluation tool for the antioxidant activities of plants. The hydromethanolic leaf extract of SL possesses antioxidant activity which is concentration-dependent, as with the reference drug (vitamin C). The antioxidant result was consistent with reports of previous studies such as [4].

Oxidative stress is a contributing factor in the pathogenesis of malaria and has been implicated in the severity and complication of malaria fever [34]. Antioxidants are scavengers of radical oxidative species (ROS) and have been found to be effective in the management of malaria infection. It is suggested that there is a positive relationship between antimalarial and antioxidant effects [35, 36]. Artemisinin is an effective antimalarial drug, which was recommended by WHO and it is stipulated that its mechanism of activity is free radical formation for the destruction of *Plasmodium*, antioxidant effect. Reference [17] documented the relationship between oxidative stress and malarial-related systemic complications. The antioxidant effect of hydromethanolic leaf extract of SL may include any one or two of the following mechanisms: free radicles scavenger or breaking of free radical chain and singlet oxygen quenching or reducing effect by electron donation [37]. The data obtained from this study are consistent with the previously reported outcome [38] which also report concentration-dependent DPPH scavenging activity. The reducing power and free radical mopping effect of SL extract suggested the possibility of the plant extract having the ability to ameliorate the effect of malaria and possibly prevent malaria-associated complications.

The chromatographic fingerprint of hydromethanolic leaf extract of SL was done employing standard phytochemical analysis methods and HPLC. The HPLC is an emerging technology that aids in the identification and quantification of the contents of a mixture of compounds. Its application in the standardization of crude plant extracts and herbal preparations has been reported by several authors [39]. The analysis of SL leaf extract using reference standards as marker compounds (flavonoid compounds) gave good separation revealing the presence of gallic acid, rutin, catechin, quercetin, kaempferol, and naringenin. These compounds are flavonoids which confirmed the phytochemical analysis result obtained. They could serve as quality control, identification, and preliminary standardization profile for the plant. Twenty-nine flavonoids were detected from the root extract of SL using the GC-MS technique and reported by Ikewuchi et al. [40]. The metabolomic profile of two other species of *Sansevieria* indicated the presence of the flavonoids identified in the present student as reported by El-Hawary et. al. [41].

Sa*nsevieria liberica* contains alkaloids, carotenoids, flavonoids, saponins, sterols, terpenes, and tannins in the preliminary assay carried out. However, anthraquinone was absent. The presence of the named secondary metabolites is in line with the reports of some other researchers' reports [42–44]. Adelanwa and Ismail Habibu [42], however, reported also the absence of anthraquinones, tannins, and alkaloids in methanol extract of the leaf. The difference may be due to the solvent used or site of collection or technical mistakes.

Phytochemical compounds such as alkaloids are commonly implicated in the antiplasmodial activity of many plants [45–48]. Terpenes or terpenoids have been identified as active antiprotozoal and antimalarial agents in many pharmacological studies [47, 49–51]. Flavonoids were also detected, and studies reveal that flavonoids give significant antiparasitic activities against different parasite strains of malaria, trypanosome, and leishmania [52–55]. Flavonoids and other phenolic compounds also have been documented to possess antioxidant potentials [46]. These phytochemical compounds which were detected may be acting singly or in synergy with one another to exert the observed antiplasmodial activity of *S. liberica*.

## 7. Conclusion

This investigation showed that *S. liberica* contains bioactive compounds that possibly possess an antimalarial effect in *in vivo* models. Thus, the traditional use of the plant in herbal preparation for malarial fever may have scientific backing. The antioxidant status properties may ameliorate the complications of malaria infection. The presence of the identified flavonoids confirms the antioxidant potential of the plant and may be used for identification and/or standardization fingerprint.

## Figures and Tables

**Table 1:** Curative effect of 90% methanolic leaf extract of SL in established infection (Rane assay).

Drug	Dose mg/kg	Parasitemia	Day 3	Day 4	Day 5	Day 6	Day 7	Day 8	MST (Days)
5% DMSO	0.2 ml	% parasitemia	11.31 ± 0.28	13.49 ± 1.04	15.38 ± 0.64	18.3 0 ± 0.42	20.47 ± 0.34	16.31 ± 0.51	8.43
		% inhibition	0	0	0	0	0	0	
CQ	10	% parasitemia	11.66 ± 0.32	10.70 ± 0.84	4.38 ± 0.68	1.39 ± 0.24	0 ± 0	0 ± 0	21^∗^
		% inhibition	-3.09	20.68	71.52	92.40	100	100	
SL	100	% parasitemia	10.61 ± 0.00	11.84 ± 0.00	10.44 ± 0.00	9.01 ± 0.00	7.75 ± 0.00	6.16 ± 0.00	21^∗^
		% inhibition	6.19	12.23	31.99	50.77	62.14	71.09∗	
	200	% parasitemia	10.86 ± 0.00	12.26 ± 0.00	10.59 ± 0.00	9.04 ± 0.00	7.47 ± 0.00	5.84 ± 0.00	21^∗^
		% inhibition	3.98	9.12	31.01	50.60	63.51	72.60∗	
	400	% parasitemia	11.35 ± 0.00	12.62 ± 0.00	11.13 ± 0.00	8.81 ± 0.00	6.91 ± 0.00	7.76 ± 0.00	14.36^∗^
		% inhibition	-0.35	6.45	27.49	51.86	66.24	62.09∗	

Values for mean % parasitemia are expressed as mean ± standard error of the mean (± SEM) *n* = 5, significant at ^*∗*^*P* < 0.05 when compared with the control. SL: *Sansevieria liberica extract*; CQ: chloroquine; MST: mean survival time (days).

**Table 2:** Retention time and identification of the peaks of hydromethanolic leaf extract of SL.

Retention time	Area (mAU × s)	Area %	Amount (ppm)	Standard drugs
9.769	42.235	5.717	2.414	Gallic acid
19.291	282.132	5.925	1.671	Catechin
32.382	363.923	3.510	12.777	Rutin
36.945	1166.490	8.426	9.829	Quercetin
38.507	239.611	7.092	1.699	Kaempferol
39.866	956.425	1.970	18.846	Naringenin

**Table 3:** Retention time of component compounds of a mixture of standard flavonoids.

Retention time	Width (min)	Area (mAU × s)	Area %	Standard drugs
9.859	0.622	8745.316	24.710	Gallic acid
19.453	0.623	8437.654	23.841	Catechin
31.368	0.749	1424.134	4.024	Rutin
36.721	0.183	5933.698	16.766	Quercetin
38.100	0.168	7049.527	19.919	Kaempferol
39.050	0.155	2537.409	7.170	Naringenin

## Data Availability

The data used to support the findings of this study are included within the article.

## Supplementary Materials

Supplementary Materials The results of the chemosuppressive effect of graded doses of leaf extract of *Sansevieria liberica* on early *P. berghei* infection are shown in supple 1. The suppression of parasite growth in the three SL treatment and chloroquine groups was statistically significant (*P* < 0.001) compared to the negative control group that received the vehicle (5% DMSO).
