# Supplementary material for: Antimalarial, Antioxidant Activities and Chemoprofile of Sansevieria liberica Gerome and Labroy (Agavaceae) Leaf Extract
Source: Adv Pharmacol Pharm Sci. 2021 Dec 6;2021:9053262. doi: 10.1155/2021/9053262 (PMC8668317; doi:10.1155/2021/9053262)
Supplement: Supplementary Materials — The results of the chemosuppressive effect of graded doses of leaf extract of Sansevieria liberica on early P. berghei infection are shown in supple 1. The suppression of parasite growth in the three SL treatment and chloroquine groups was statistically significant (P < 0.001) compared to the negative control group that received the vehicle (5% DMSO). [file 9053262.f1.docx]

Supplementary materials:

| **Plant/ Drug** | **Dose (mg/kg)** | **% Parasitemia** | **% Suppression** |
| --- | --- | --- | --- |
| 5% DMSO | 0.2 ml | 11.28 ± 0.35 | 0 |
| Chloroquine | 10 | 1.20 ± 0.02 | 89.36 |
| SL | 100 | 3.53 ± 0.1963 | 68.71* |
|  | 200 | 3.3 ± 0.1273 | 70.74* |
|  | 400 | 2.808 ± 0.2065 | 75.09* |

**Suppl 1: Suppressive effect of 90% methanolic leaf extract of SL in early infection of *Plasmodium berghei***
